# Identification of novel, *in vivo* active Chk1 inhibitors utilizing structure guided drug design

**DOI:** 10.18632/oncotarget.5929

**Published:** 2015-09-30

**Authors:** Andrew J. Massey, Stephen Stokes, Helen Browne, Nicolas Foloppe, Andreá Fiumana, Simon Scrace, Mandy Fallowfield, Simon Bedford, Paul Webb, Lisa Baker, Mark Christie, Martin J. Drysdale, Mike Wood

**Affiliations:** ^1^ Vernalis Research, Granta Park, Cambridge, UK; ^2^ Akranim Ltd, Maidstone, UK; ^3^ Horizon Discovery, Cambridge Research Park, Waterbeach, Cambridge, UK; ^4^ Cancer Research UK Beatson Institute, Garscube Estate, Bearsden, Glasgow, UK

**Keywords:** Chk1, V158411, fragment, drug discovery, colon cancer

## Abstract

Chk1 kinase is a critical component of the DNA damage response checkpoint especially in cancer cells and targeting Chk1 is a potential therapeutic opportunity for potentiating the anti-tumor activity of DNA damaging chemotherapy drugs. Fragment elaboration by structure guided design was utilized to identify and develop a novel series of Chk1 inhibitors culminating in the identification of V158411, a potent ATP-competitive inhibitor of the Chk1 and Chk2 kinases. V158411 abrogated gemcitabine and camptothecin induced cell cycle checkpoints, resulting in the expected modulation of cell cycle proteins and increased cell death in cancer cells. V158411 potentiated the cytotoxicity of gemcitabine, cisplatin, SN38 and camptothecin in a variety of p53 deficient human tumor cell lines *in vitro*, p53 proficient cells were unaffected. In nude mice, V158411 showed minimal toxicity as a single agent and in combination with irinotecan. In tumor bearing animals, V158411 was detected at high levels in the tumor with a long elimination half-life; no pharmacologically significant *in vivo* drug-drug interactions with irinotecan were identified through analysis of the pharmacokinetic profiles. V158411 potentiated the anti-tumor activity of irinotecan in a variety of human colon tumor xenograft models without additional systemic toxicity. These results demonstrate the opportunity for combining V158411 with standard of care chemotherapeutic agents to potentiate the therapeutic efficacy of these agents without increasing their toxicity to normal cells. Thus, V158411 would warrant further clinical evaluation.

## INTRODUCTION

DNA damaging cytotoxic chemotherapeutic agents and ionizing radiation are the mainstay of current cancer treatment regimens. These therapies are effective, especially when administered in combinations, against a wide variety of neoplasms and are likely to remain the standard of care for cancer treatment in the foreseeable future. Due to their mechanism of action, these agents have limitations which restrict their overall effectiveness. As they target DNA, they are effective against any cell especially those actively replicating. Therefore, such cytotoxic chemotherapeutic agents lack tumor cell specificity. Administration is usually at the maximum tolerated dose (MTD) resulting in a narrow therapeutic index and toxicity to normal tissue, especially those within an actively dividing cell component such as the gastrointestinal tract and the hematological system. Acquired or intrinsic resistance can further limit the usefulness of these agents, making many patients' tumors refractory to the drug. Multiple mechanisms can contribute to acquired resistance including reduced cellular levels of active drug (through increased metabolism, detoxification or active efflux), increased DNA repair, loss of p53 or attenuation of apoptotic signaling.

Cytotoxic chemotherapeutic agents such as cisplatin, irinotecan or gemcitabine induce DNA damage, especially DNA strand breaks, through multiple mechanisms (e.g. topoisomerase inhibition, direct DNA alkylation or reduction of deoxyribonucleotides). These strand breaks activate cell cycle checkpoints resulting in cell cycle arrest and activation of DNA repair [1, 2]. Cell cycle checkpoints exist to protect the fidelity of DNA replication and division, and ensure the correct timing of cell cycle events. As DNA cannot be replaced, these pathways are critical in protecting genomic integrity and preventing the onset of cancer. Checkpoints exist at multiple phases of the cell cycle and can be activated during the G1-, S- or G2-phases in response to DNA damage [3–6]. In addition, the mitotic checkpoint is activated by improper chromosome attachment to a bipolar spindle, to protect against inaccurate chromosome segregation and aneuploidy [7]. In mammalian cells, the key effector proteins are p53 and the checkpoint kinases Chk1 and Chk2 [3, 6]. A large proportion of human cancers are defective for the p53 pathway in some form, thereby lacking a functional G1 checkpoint. Therefore, these human tumors are highly reliant on the Chk1 kinase to protect them in response to DNA damage [4, 5].

DNA damage sensing by either the Mre11 complex (Mre11, Rad50 and Nbs1), that recognizes double strand breaks, or the Rad17 and Rad9-Hus1-Rad1 complex that recognizes replication stress, activate the central transducing kinases ATM and ATR. In turn, these kinases directly activate the effector kinases Chk1 and Chk2. Chk1 is predominantly activated by three phosphorylation events; on S317 and S345 by ATR [8, 9] and autophosphorylation on S296 [10]. Chk1 and Chk2 negatively regulate the Cdc25 family of phosphatases thereby preventing cell cycle progression as well as directly modulating repair proteins, resulting in increased lesion repair [11]. Biochemical and genetic studies have demonstrated Chk1 to be indispensable for the S- and G2-M checkpoints [4, 12].

Chk1 inhibition, therefore, represents a novel therapeutic strategy to increase the cytotoxicity of DNA-damaging chemotherapeutic drugs in p53 pathway defective cancers [13, 14]. Abrogation of the Chk1 dependent checkpoint should result in increased tumor cell death. This approach should increase the therapeutic index of a chemotherapeutic drug, as normal cells should remain protected by their functional p53 pathway. This approach is under evaluation with several small molecule inhibitors of Chk1 in Phase I (AZD7762, PF-477736, GDC-0425 and GDC-0575) or Phase II (LY2603618 and MK-8776 (SCH 900776)) trials in combination with gemcitabine, irinotecan, pemetrexed and cytarabine [15–18]. Chk1 may also function in the mitotic spindle checkpoint [19] and the Chk1 inhibitor PF-477736 has demonstrated potentiation of docetaxel-induced efficacy in xenografts [20]. The status of these clinical trials has been summarized [14, 21].

The present work describes the use of structure-guided fragment evolution [22] in the identification and subsequent pre-clinical characterization of V158411, a novel, potent, selective small molecule inhibitor of Chk1. V158411 potently inhibited Chk1 and Chk2 kinases and abrogated DNA damage induced S- and G2-phase checkpoints. The *in vitro* cytotoxicity of gemcitabine, cisplatin, SN38 and camptothecin was potentiated by V158411 in p53 deficient, but not in p53 proficient, human tumor cell lines. *In vivo*, V158411 exhibited good pharmacokinetic properties, showed minimal toxicity and potentiated the anti-tumor activity of irinotecan in human tumor xenograft models without additional systemic effects. These results demonstrate the potential of combining V158411 with standard of care chemotherapeutic agents to enhance the therapeutic efficacy of these agents, without increasing their toxicity to normal cells.

## RESULTS

### Identification and elaboration of a fragment core using structure guided drug design to generate a novel series of potent Chk1 inhibitors

**Figure 1:**
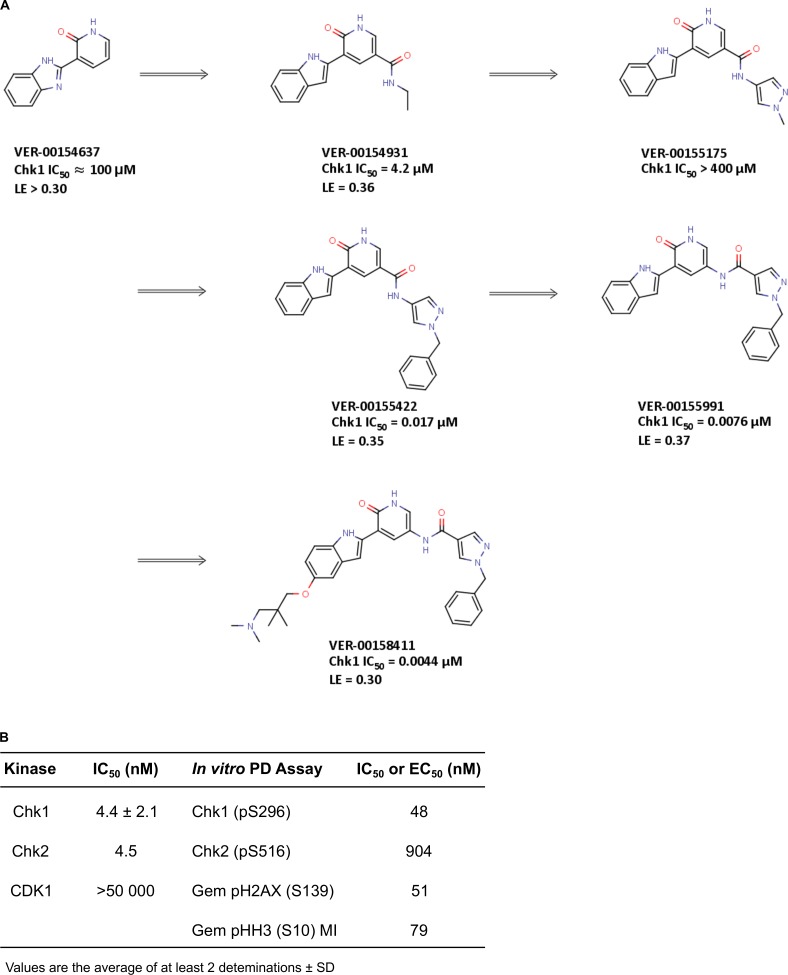
Identification of V158411 by fragment elaboration utilizing structure guided drug design **A.** Elaboration of a weak binding but ligand efficient fragment (VER-154637) into a novel series of Chk1 kinase inhibitors. **B.** *In vitro* activity of V158411.

**Figure 2:**
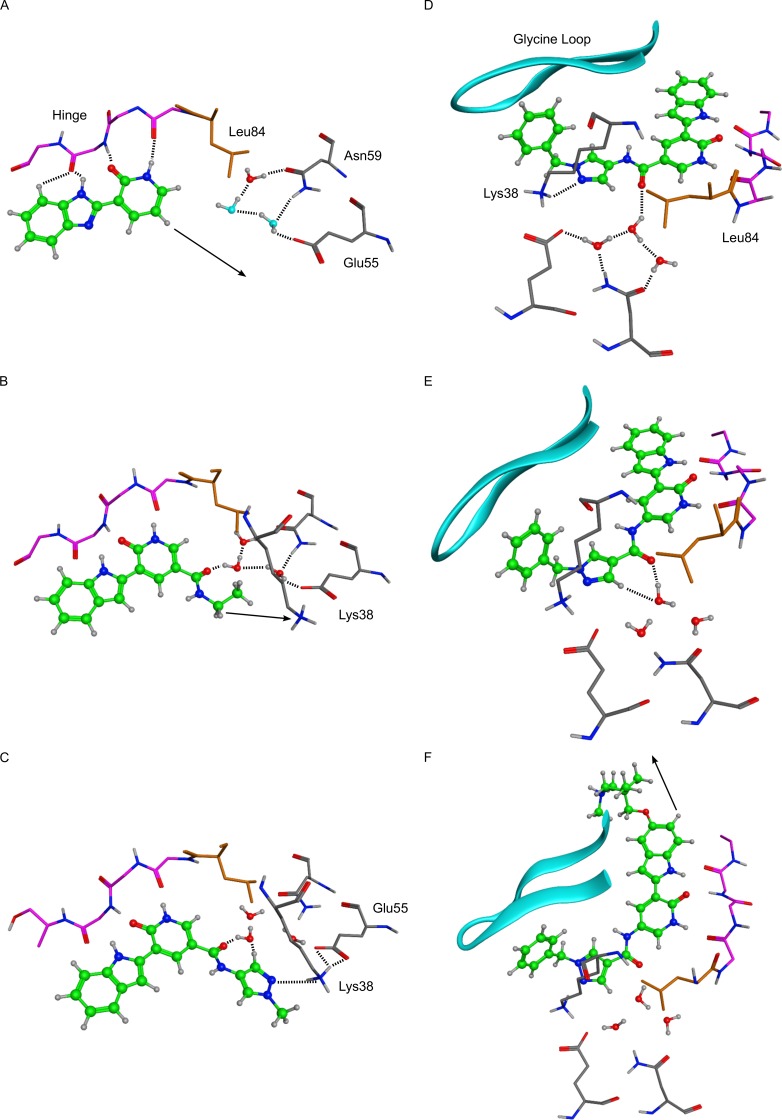
X-ray crystal structures of key molecules in evolution of VER-154637 to V158411 Hydrogen atoms were added to the X-ray coordinates with the software MOE, and only selected hydrogens are shown. Dotted lines indicate inferred hydrogen-bond interactions, and arrows indicate vectors used for structure-guided chemical elaboration. Key amino acids and structural features are indicated. In panel A, the two water molecules with light blue oxygens were modelled by analogy with the three conserved water molecules observed in most Chk1 X-ray structures. **A.** VER-154637. **B.** VER-154931. **C.** VER-155175. **D.** VER-155422. **E.** VER-155991. **F.** V158411 (PDB ID: 5DLS).

The commercially available benzimidazole-1H-pyridin-2-one fragment (VER-154637) was identified as a weak, but ligand efficient, binder to the Chk1 kinase (Figure 1A, IC_50_ ≈ 100 μM, LE > 0.30). The X-ray structure of VER-154637 bound to the ATP-binding site of Chk1 showed that the two rings of the fragment were coplanar and made the expected hydrogen-bonds with the hinge motif of the kinase domain (Figure 2A). The benzimidazole core was replaced by an isomorphous indole ring, with the possible advantage that there is only one possible tautomer for the polar hydrogen of the indole ring. The indolyl-pyridone core bound to Chk1 like the parent fragment, and formed the basis for subsequent molecular modeling and compound elaboration.

The crystal structure (Figure 2A) demonstrated that substituents added at the pyridone position 6 (Figure 1A) would likely clash with the Chk1 gatekeeper residue Leu84. Conversely, the indole vectors C_5_─H and C_6_─H point towards a solvent-exposed part of the binding-site, with limited opportunities for tight contacts with the protein. In addition, computational conformational analysis suggested that derivatization from the indole position 3 or the pyridone position 4 would sterically twist those rings out of coplanarity, in turn disrupting hydrogen-bonds to the kinase hinge. Thus, the initial chemistry efforts concentrated on growing the fragment at the pyridone position 5. The corresponding C_5_─H vector was in the vicinity of the three buried water molecules, which are usually conserved in X-ray structures of Chk1. Molecular modeling suggested that the well-defined orientation of the Chk1 side-chains and backbone around these water molecules probably results in a particular predominant hydrogen-bond network between the waters and residues Glu55, Asn59, Val68, Asp148 and Phe149 (Figure 2A). It implies a strong orientational preference for these water molecules, such that the water closest to the ligand would act mostly as a hydrogen-bond donor towards the compound. Modeling suggested that an amide linker grafted on the pyridone position 5 would offer its carbonyl group as hydrogen-bond acceptor complementary to the hydrogen-bond donor character of the contacting water (Figure 2A–2B). This prediction was born out crystallographically, following the introduction of a small amide at the C-5 pyridin-2-one (VER-154931, Figure 2B). VER-154931 was a low μM inhibitor which maintained the ligand efficiency of the parent fragment. The amide nitrogen offered the opportunity to grow towards the largely buried and structurally restrained side-chain amino group of Lys38 (Figure 2B). To this end, the amide linker was extended with several hydrogen-bond-accepting groups of approximately the desired length. A methylated pyrazole was shown to bridge to Lys38 by X-ray crystallography, although with a disappointing affinity (VER-155175, Figure 2C). Yet, benzylation of the pyrazole led to a potency breakthrough (VER-155422, IC_50_ 0.017 μM, LE 0.35). The X-ray structure of VER-155422 bound to Chk1 (Figure 2D) showed that the benzyl tucks underneath the flexible glycine loop, burying the apolar benzyl away from water, which presumably explains the associated affinity gain. It was then noted that reversing the intramolecular direction of the amide linker could maintain its hydrogen-bond with the conserved water, while also keeping the desired compound length for binding to Lys38. Inversion of the amide linker in VER-155991 (Figure 2E) gave a 2-fold increase in potency (IC_50_ 0.0076 μM).

Much of the subsequent medicinal chemistry concentrated on improving the compounds physico-chemical and ADMET properties. This was done by varying substituents at the solvent-exposed 5 position of the indole ring which, from a structural point of view, can tolerate a broad range of substituents, largely unhindered from specific interactions with the protein. No attempt was made to design compounds which would be selective for Chk1 over Chk2. This led to V158411 (Figure 2F), which had the desired kinase selectivity profile and showed promising biological activity.

### V158411 is a potent and selective inhibitor of checkpoint kinases

V158411 potently inhibited the kinase activity of full length Chk1 and Chk2 with IC_50_s of 4.4 and 4.5 nM respectively and, importantly, was more than 10 000-fold selective for CDK1 (Figure 1B). Selectivity against the cell cycle kinase CDK1 is considered critical for the pharmacological activity of Chk1 inhibitors as inhibiting it would prevent the G2 checkpoint abrogation and entry into mitosis; critical for the potentiation of cytotoxic DNA damage [23]. Against a panel of 386 kinases in a wide panel binding assay, V158411 inhibited the activity of one kinase (Chk1) in the range 99-100%, three kinases 90-99% and 19 kinases 65-90% at 50 nM (Supplementary Table 1). V158411 demonstrated little activity as a single agent against a panel of human cancer cell lines inhibiting cell proliferation with GI_50_s between 0.50 and 9.5 μM (Supplementary Table 2).

### V158411 inhibits Chk1 autophosphorylation and abrogates DNA damage induced cell cycle arrest

**Figure 3:**
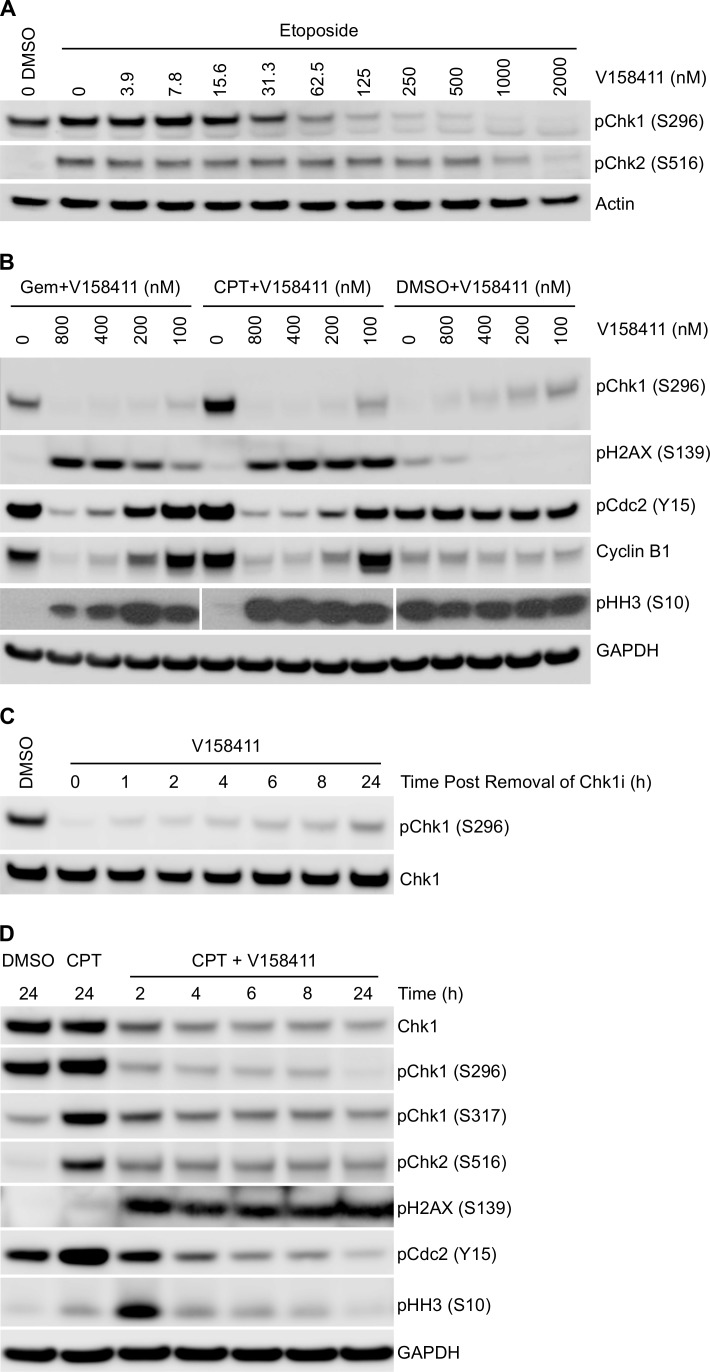
Effect of V158411 on etoposide, gemcitabine and camptothecin induced DNA damage checkpoint and cell cycle proteins **A.** HT29 cells were exposed to increasing concentrations of V158411 for 1 hour followed by 25 μM etoposide for a further 5 hours. Auto-phosphorylation of Chk1 and Chk2 was determined by immunoblotting. **B.** HT29 cells were treated with gemcitabine (25 nM) or camptothecin (50 nM) for 16 hours followed by increasing doses of V158411 for a further 24 hours. **C.** HT29 cells were treated with 500 nM V158411 for 1 hour, media removed and replaced with drug free. Cells were harvested at the indicated time points post the removal of V158411. **D.** HT29 cells were treated with 0 or 100 nM camptothecin for 16 hours followed by V158411 for 2 - 24 hours. After the indicated time, media was removed and replaced with drug free media. All cells were harvested 24 hours after the initial addition of V158411. Protein expression was characterized by immunoblotting as described in materials and methods.

The ability of V158411 to modulate protein markers of cell cycle status and DNA damage were evaluated. In the absence of DNA damage, low levels of Chk1 phosphorylated on S296 or Chk2 phosphorylated at S516 are detectable, but this is rapidly increased in HT29 cells treated with etoposide (Figure 3A). V158411 reduced pChk1 (S296) and pChk2 (S516) levels in a concentration dependent fashion in cells treated with etoposide with IC_50_s of 48 nM for Chk1 and 904 nM for Chk2; i.e. a cellular selectivity of 19-fold for Chk1 versus Chk2. Inhibition of Chk1 following DNA damage leads to checkpoint failure, an increase in DNA strand breaks and subsequent activation of H2AX *via* phosphorylation on S139, a marker of DNA strand breaks [24, 25]. Using an ELISA assay specific for pH2AX (S139), V158411 in combination with gemcitabine induced H2AX phosphorylation with an EC_50_ of 51 nM, in close agreement with its ability to suppress Chk1 auto-phosphorylation. Treatment of HT29 cells with either gemcitabine or camptothecin likewise led to an increase in pChk1 (S296) that could be effectively blocked by V158411 in a concentration dependent fashion (Figure 3B) with complete ablation achieved at concentrations of V158411 between 100 and 200 nM. Abrogation of Chk1 phosphorylation correlated with increased phosphorylation of Histone H2AX on S139. Maximal induction of H2AX phosphorylation, as determined by western blotting, occurred at concentrations between 100 and 200 nM V158411. In cell culture, V158411 appeared to exhibit a long residency time on the Chk1 protein. Treatment of HT29 cells with V158411 for 1 hour resulted in Chk1 inhibition (as measured by phosphorylation of Chk1 at S296) for at least 24 hours after the removal of V158411 from the culture media (Figure 3C). Treatment of HT29 cells with camptothecin followed 16 hours later by a 2 hour pulse of V158411 followed by a 22 hour washout period resulted in the same degree of Chk1 inhibition and induction of pH2AX (S139) as a continual exposure to V158411 (Figure 3D) for 24 hours. DNA damage induced by cytotoxic drugs such as gemcitabine or camptothecin activates cell cycle checkpoints and cell cycle arrest. These changes are detectable by measuring the expression levels and phosphorylation status of cell cycle proteins. The arrest of cells in S- or G2/M phase by gemcitabine and camptothecin was evidenced by the increase in Cdc2 Y15 phosphorylation and cyclin B1 levels and a decrease in Histone H3 phosphorylation (pHH3 (S10) (Figure 3B). V158411 was able to alleviate this cell cycle block decreasing Cdc2 phosphorylation and cyclin B1 levels as wells as increasing pHH3 (S10) in a concentration dependent manner. Abrogation of these cell cycle blocks correlated closely with decreased Chk1 S296 phosphorylation and increased H2AX phosphorylation (Figure 3B). Similar results were obtained in Colo205 and SW620 cells treated with gemcitabine or camptothecin (Supplementary Figure 1). In these two cell lines, V158411 reduced gemcitabine and camptothecin induced S296 Chk1 phosphorylation and increased S139 H2AX phosphorylation. These changes correlated with a decrease in Cdc2 Y15 phosphorylation and an increase in pHH3 (S10). These results provide strong evidence that inhibition of Chk1 with V158411 results in abrogation of cell cycle arrest induced by DNA damaging agents and increased DNA damage.

The ability of V158411 to abrogate gemcitabine induced S-phase arrest was further characterized using a checkpoint abrogation assay. Cells were pre-treated with gemcitabine for 16 hours followed by increasing concentrations of V158411 in the presence of nocodazole, an inhibitor of microtubule polymerization, for a further 24 hours. Mitotic cells were then scored for pHH3 (S10) expression. p53 defective HT29 cells exposed to gemcitabine, which induces arrest in S-phase, were negative for pHH3 (S10) staining. V158411 treatment abrogated the gemcitabine induced S-phase arrest allowing cells to progress through into mitosis where they were trapped by nocodazole. These cells stained positive for pHH3 and are indicative of checkpoint abrogation. V158411 abrogated the gemcitabine induced S-phase arrest with an EC_50_ of 79 nM and maximal abrogation at around 300 nM (Figure 1B).

### V158411 potentiates cytotoxic agents in p53 defective cancer cell lines

**Figure 4:**
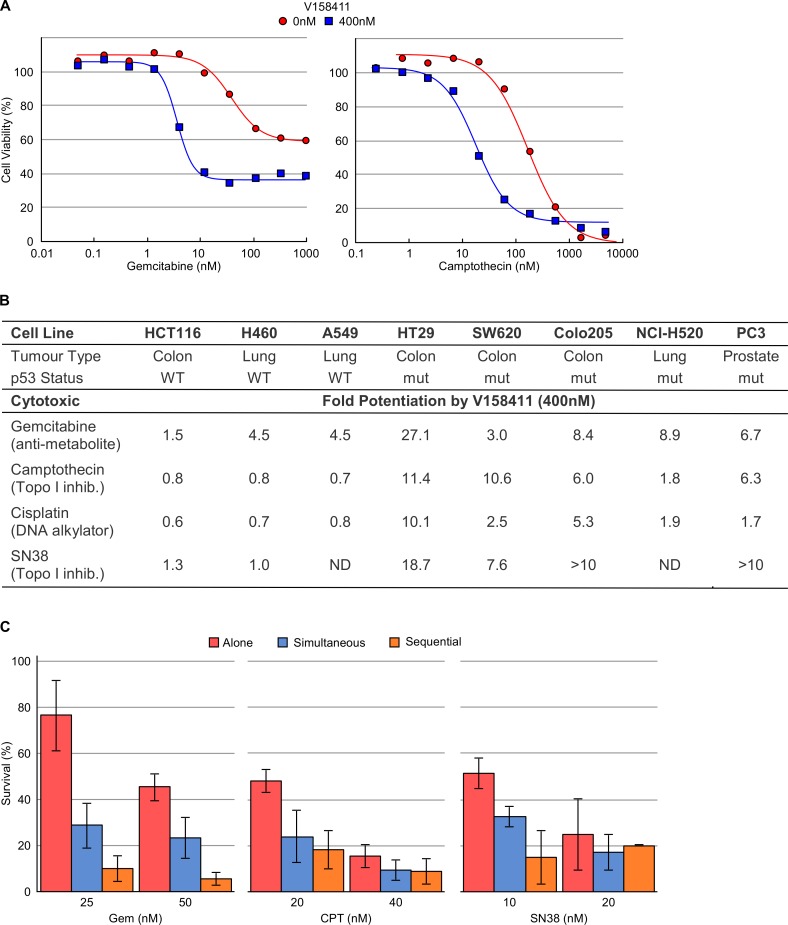
V158411 potentiates gemcitabine, camptothecin, cisplatin and SN38 cytotoxicity *in vitro* **A.** Curves representing the antiproliferative effect of gemcitabine or camptothecin in HT29 cells in combination with 0 or 400 nM V158411. **B.** *In vitro* potentiation of gemcitabine, camptothecin, cisplatin or SN38 cytotoxicity by 400 nM V158411 in p53 proficient or deficient cell lines. Potentiation factor was calculated by IC_50(cytotoxic agent alone)_ / IC_50(combination treatment)_. **C.** Colony formation assays in HT29 cells following treatment with gemcitabine, camptothecin or SN38 alone for 24 hours, in combination with 300 nM V158411 for 24 hours or for 24 hours followed by 300 nM V158411 for a further 24 hours. Media was then replaced with drug free and colonies stained after 14 days. Values are the average of 3 independent determinations ± SD.

The ability of V158411 to potentiate the cytotoxicity of a variety of chemotherapeutic drugs was assessed across a panel of cell lines. V158411 potentiated the growth inhibitory activity of a wide variety of chemotherapeutic agents across a panel of p53 defective but not p53-proficient cell lines (Figure 4A and 4B). The agents tested are representative of chemotherapeutic agents currently in clinical use with differing modes of action including antimetabolites (gemcitabine), topoisomerase I inhibitors (camptothecin and SN38) and platinating agents (cisplatin). The concentration of V158411 used to potentiate the cytotoxic activity of these agents had no activity as a single agent (Supplementary Table 2). As has been seen with other Chk1 inhibitors [15, 16, 26, 27], the most robust potentiation was observed with gemcitabine. With gemcitabine, V158411 decreased the overall cell viability, in addition to reducing the GI_50_ of gemcitabine.

The ability of V158411 to increase the cytotoxicity of gemcitabine, camptothecin or SN38 was confirmed using colony formation assays. Treatment of HT29 cells with gemcitabine, camptothecin or SN38 in combination with V158411 significantly increased the fraction of cells killed by all three agents (Figure 4C). Comparison of two different dosing regimens, co-dosing or cytotoxic agent followed by V158411, indicated little schedule dependence for camptothecin or SN38. However, for gemcitabine, schedule dependence was observed. Sequential administration of V158411 24 hours after gemcitabine appeared more effective than co-treatment.

### *In vivo* pharmacokinetics of V158411 in preclinical species

**Figure 5:**
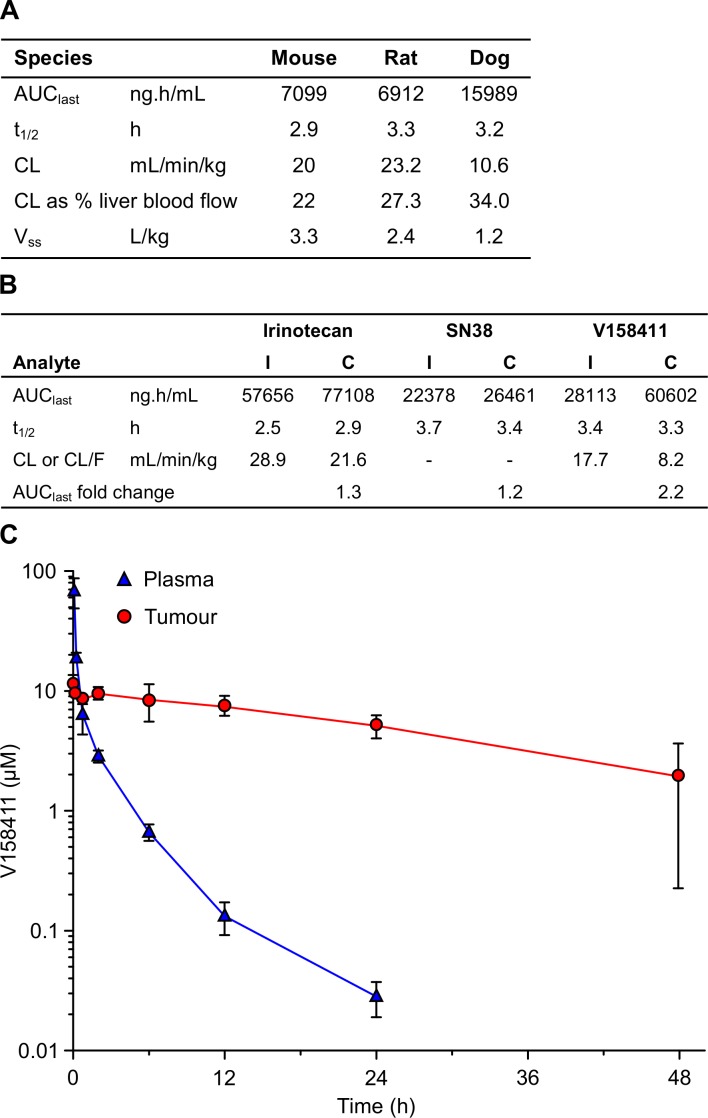
*In vivo* pharmacokinetic properties of V158411 **A.** Summary of V158411 pharmacokinetics in the mouse, rat and dog following 10 mg/kg IV administration. Mouse data is from a composite profile with 3 mice per time point, rat and dog data are the mean of individual pharmacokinetic profiles (*n* = 3 or 4). **B.** Summary of V158411 and irinotecan pharmacokinetics in the mouse following single administration (I) of 30 mg/kg IV V158411 or 100 mg/kg IP irinotecan, or combination administration (C) of 100 mg/kg IP irinotecan followed 2 hours later by 30 mg/kg IV V158411. Data is from composite profiles with 3 mice per time point. **C.** Pharmacokinetics of V158411 in HT29 tumor bearing mice following 30 mg/kg IV administration in plasma and tumor. *Points*, mean for three mice per time point; *bars*, SD. Drug concentrations were determined as detailed in Materials and Methods.

Administration of V158411 to three pre-clinical species resulted in comparable plasma pharmacokinetics following a single IV bolus of 10 mg/kg. In all three species V158411 demonstrated low plasma clearance and moderate volumes of distribution resulting in reasonably long plasma half-lives (Figure 5A). The pharmacokinetics of irinotecan, its' active metabolite SN38, and V158411, were determined in mice following single doses and in combination. The combination dose regimen mimicked an efficacious dosing regimen from xenograft studies *in vivo*. The combination of 100 mg/kg intraperitoneal irinotecan, followed 2 hours later by 30 mg/kg intravenous V158411, had no major effect on the plasma pharmacokinetics of irinotecan and SN38 (Figure 5B). Conversion of irinotecan to SN38 was not affected by the combination dosing. Co-administration of irinotecan and V158411 resulted in a small decrease in the clearance and subsequent increase in the exposure of V158411 (2.2 fold increase in AUC_last_). The mechanism underlying this effect is unknown. A pharmacokinetic study in HT29 tumor bearing mice demonstrated that V158411 rapidly distributes out of the plasma into the tumor. Following IV administration at 30 mg/kg, V158411 has a low plasma clearance (24 mL/min/kg) and a moderate volume of distribution (2.5 L/kg), resulting in a long half-life (4.1 h). V158411 rapidly distributes out of the blood and into tissues: tumor exposure was considerably higher than plasma as quantified by a tissue/plasma AUC_0-24h_ ratio of 4.7. In the tumor, V158411 has a considerably longer elimination half-life (22 hours) than observed in the plasma (Figure 5C).

### V158411 potentiates the anti-tumor activity of irinotecan in human xenograft models

**Figure 6:**
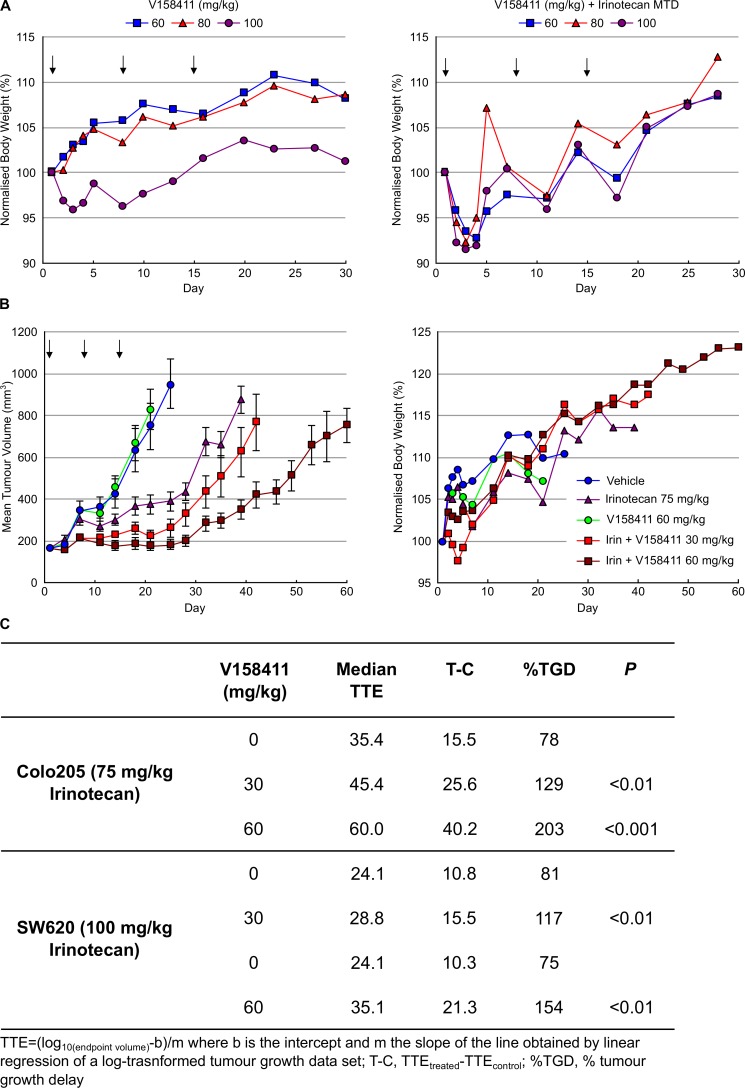
V158411 potentiates irinotecan cytotoxicity in human tumor xenografts **A.** The MTD dose of V158411 as a single agent (left) or in combination with MTD irinotecan (right) was determined in female *nu/nu* mice. V158411 was administered IV q7d for 3 weeks as a single agent or 2 hours after 100 mg/kg IP (MTD) irinotecan. Values are the mean normalized body weight of five animals. **B.** *nu/nu* mice bearing established Colo205 xenografts were treated q7d for 3 cycles with vehicle, 75 mg/kg IP irinotecan, 60 mg/kg IV V158411 or the combination of 75 mg/kg irinotecan IP followed by 30 mg/kg or 60 mg/kg V158411 IV 2 hours later. Tumor size and body weight were determined as described in the materials and methods. **C.** Summary of *in vivo* responses to irinotecan plus V158411 combination therapy.

Based on the pharmacokinetic profile of V158411 and the need to combine with a cytotoxic chemotherapeutic agent, a once weekly IV dosing schedule was initially selected. The maximum tolerated dose (MTD) of V158411 as a single agent was determined following administration of 60 to 100 mg/kg q7d for 21 days to female nude mice in groups of five animals (Figure 6A). V158411 had no effect on body weight loss, and no treatment related deaths occurred at doses up to 100 mg/kg. Higher doses could not be tested due to compound solubility and dosing volume limitations. V158411 caused some acute, dose-related effects (flushing, tachycardia, exophthalmos) that were self-limiting and could be minimized by administering the compound by slow infusion. We further confirmed that V158411 did not increase the systemic toxicity of the cytotoxic agent irinotecan in *nu/nu* mice. Irinotecan is a pro-drug of SN38, which inhibits the activity of topoisomerase I, and a semi-synthetic analogue of camptothecin and is currently used clinically for the treatment of colon cancer. Administration of the MTD of irinotecan in combination with doses of V158411 up to 100 mg/kg did not result in increased irinotecan toxicity (Figure 6A).

The ability of V158411 to potentiate the anti-tumor efficacy of irinotecan *in vivo* was assessed in mouse colon carcinoma xenograft models. Colo205 tumor bearing mice were treated with combinations of irinotecan and V158411 once weekly for 3 weeks until the mean tumor volume reached 750 mm^3^ or 60 days post the first treatment had elapsed. In the absence of any treatments, the median time to endpoint (TTE) was 19.8 days (Figure 6B). Treatment with 60 mg/kg V158411 monotherapy was inactive with a median TTE of 22.3 days. In comparison, 75 mg/kg irinotecan monotherapy significantly reduced tumor growth compared to vehicle alone (*P* < 0.001). The median TTE was extended to 35.4 days equating to a 79% tumor growth delay (TGD). Addition of 30 mg/kg or 60 mg/kg V158411 2 hours post 75 mg/kg irinotecan to the treatment regimen significantly decreased tumor burden and slowed time to endpoint compared to irinotecan therapy alone (Figure 6B). In the mice treated with 30 mg/kg V158411, the median TTE was extended to 45.4 days corresponding to a 129% TGD (*P* < 0.01). At 60 mg/kg V158411, the median TTE was further extended to 60 days with a 203% TGD (*P* < 0.001, Figure 6C). There was no additional systemic toxicity associated with the combination therapy with no animal deaths observed and a mean body weight nadir on day 4 of −2.7% (Figure 6B).

The potentiation of irinotecan anti-tumor efficacy by V158411 was confirmed in a SW620 colorectal mouse xenograft model. This model grows more rapidly than the Colo205 model, reaching a median tumor volume of 1500 mm^3^ (the endpoint in these studies) in 13.3 days. V158411 did not inhibit tumor growth in this model when administered as a monotherapy. Administration of irinotecan on a once weekly for 3 weeks schedule significantly reduced SW620 tumor growth with a median TTE of 24.1 days (*P* < 0.001) equating to a TGD of 81% and 75% respectively (Figure 6C). V158411, when administered at 30 or 60 mg/kg 2 hours after irinotecan significantly increased the tumor growth delay compared to irinotecan therapy alone. Median TTEs were increased to 28.8 and 35.1 days for 30 and 60 mg/kg V158411 respectively (Figure 6C) equating to 117 and 154% TGD (*P* < 0.01). Irinotecan and the combination treatment were again well tolerated in the SW620 animal model. The mean body weight nadir for irinotecan alone was −8.6% on day 4 and −13.3% on day 5 for the combination group with 60 mg/kg V158411. No treatment-related animal deaths were observed in any treatment group. In summary, a single IV dose of V158411 administered 2 hours after irinotecan significantly increased the anti-tumor activity of irinotecan in two different human colon cancer xenograft models without increasing the systemic toxicity of irinotecan.

## DISCUSSION

Chk1 is a key component of the DNA damage response pathway and is activated in response to intrinsic DNA damage induced by normal cellular processes such as replication fork collapse or in response to DNA damage induced by cytotoxic chemotherapeutic agents. Chk1 inhibitors represent a new molecularly targeted class of chemopotentiators that could increase the therapeutic activity of standard of care cytotoxic drugs whilst sparing the normal tissues from increased systemic toxicity. This hypothesis is currently being tested in the clinic with the IV inhibitors LY2603618 (Phase II) and MK-8776 (Phase II) and the oral inhibitors GDC-0425 and GDC-0575 (both Phase I) [28, 29] (http://clinicaltrials.gov/).

V158411 is a novel inhibitor of the Chk1 kinase discovered through structure-based elaboration of a fragment core designed for binding at a kinase ATP-binding site. In an *in vitro* assay against recombinant human Chk1, V158411 inhibited the phosphorylation of a substrate peptide with an IC_50_ of 4.4 nM. This is below the lower theoretical limit of the assay (6.25 nM, half enzyme concentration in the assay) suggesting that V158411 may inhibit Chk1 with greater potency than detected in this assay. Cross screening against the functionally related Chk2 kinase revealed little selectivity between Chk1 and Chk2 at the enzyme level. The clinical and therapeutic relevance of inhibiting Chk2 is still unclear [30] and Chk1 versus Chk2 selective (PF-477736 and MK-8776) [16, 17] and non-selective (AZD7762) [15] inhibitors have undergone clinical evaluation. Against a wide panel of diverse kinases, V158411 exhibited > 100-fold selectivity for Chk1 over the majority of the kinases in the panel at pharmacologically relevant doses. Selectivity against the cell cycle kinase CDK1 (> 10 000-fold selective) is considered critical for the pharmacological activity of Chk1 inhibitors as inhibiting it would prevent the G2 checkpoint abrogation and entry into mitosis; critical for the potentiation of cytotoxic DNA damage [23]. V158411 exhibited very little single agent activity against a diverse panel of human cancer cell lines inhibiting proliferation with GI_50_s in the range 0.5 to 9.5 μM, further evidence of the selectivity of V158411 for Chk1 kinase.

Chk1 and Chk2 undergo autophosphorylation on serine 296 and on serine 516 respectively in response to DNA damage [9, 31, 32]. In HT29 colon cancer cells, V158411 blocked etoposide induced autophosphorylation of Chk1 with an IC_50_ of 48 nM and Chk2 with an IC_50_ of 904 nM suggesting that the apparent selectivity of V158411 for Chk1 over Chk2 is closer to 19-fold in cells. This difference in selectivity cannot be attributed to differences in the ATP K_m_ and is likely due to the kinases being present in dynamic, multi-protein complexes. This highlights the risk of relying solely on isolated enzyme assays to determine kinase selectivity.

Abrogation of gemcitabine induced cell cycle arrest was determined using a high content assay to measure mitotic cells following trapping with nocodazole and flow cytometry. In both assays, V158411 inhibited gemcitabine induced checkpoint activation in a concentration range that correlated closely with inhibition of Chk1 kinase activity (as determined by inhibition of Chk1 S296 phosphorylation). Abrogation of DNA damage induced cell cycle checkpoints resulted in increased DNA damage as determined by an increase in H2AX phosphorylation on S139. This increase in H2AX phosphorylation correlated with Chk1 kinase inhibition and DNA damage induced checkpoint abrogation. The IC_50_ for inhibition of Chk1 autophosphorylation and the EC_50_ for induction of H2AX phosphorylation by V158411 was around 10-fold lower in HT29 cells than the single agent GI_50_ suggesting that achieving a reasonable therapeutic index should be possible. The ability of V158411 to potentiate the *in vitro* cytotoxicity of a range of cytotoxic agents of differing mechanism of actions was determined in a panel of p53 wild type and mutant cancer cell lines. Substantial potentiation of the anti-tumor activity of gemcitabine (anti-metabolite), camptothecin and SN38 (topoisomerase I inhibitors), and cisplatin (DNA alkylator) by V158411 was observed in all five p53 mutant cancer cell lines but not in the three p53 wild type cell lines. Whilst we have been unable to conduct studies on isogenic cell lines, these results suggest that the enhanced cytotoxicity observed with V158411 will be dependent on the p53 status. Previous studies have suggested p53 mutant status to be important for the overall response to combinations with some agents but does not predict synergy in all cases [26]. This further supports the notion that V158411 should have a good therapeutic index in man. As has been observed with other Chk1 inhibitors [15, 16, 26, 27] gemcitabine cytotoxicity was potentiated the greatest by V158411. Interestingly, this was the only chemotherapeutic agent where V158411 not only increased the anti-proliferative effect (as determined by a decrease in GI_50_) but reduced the viability of the cells treated with the cytotoxic agent. Whether gemcitabine is the best drug to combine with Chk1 inhibitors in the clinic still remains to be determined.

When administered *via* the intravenous route, V158411 exhibited good plasma exposure and a high volume of distribution resulting in a reasonable half-life of 4.1 hours. V158411 rapidly distributed to tumors resulting in higher exposure than in the plasma with an AUC ratio of 4.7. Selective retention of V158411 in the tumor resulted in a longer elimination half-life of 22 hours and extended tumor exposure. Comparable pharmacokinetic properties were observed across the three pre-clinical toxicology species (mouse, rat and dog) studied. In combination pharmacokinetic studies, V158411 did not alter the exposure or metabolism of either irinotecan or its active metabolite SN38 suggesting that any increased efficacy observed between irinotecan and V158411 is not due to a drug-drug interaction.

When administered either once or twice weekly as an IV bolus, V158411 showed minimal toxicity at doses up to 100 mg/kg. Doses beyond this could not be investigated due to dosing volume and V158411 solubility limitations, and the maximum tolerated dose of V158411 has yet to be found. Administration of V158411 in combination with the maximum tolerated dose of irinotecan did not affect the toxicity of irinotecan. The long tumor half-life of V158411 predicted that a single IV dose of V158411 should be sufficient to potentiate the anti-tumor activity of cytotoxic chemotherapeutic agents *in vivo*. Dose dependent potentiation of irinotecan therapy was observed in two colon cancer models. In these models, V158411 was administered as a single IV bolus 2 hours after irinotecan therapy. Further studies identified a window of between 2 and 24 hours after irinotecan as being optimal for the administration of V158411 in order to obtain therapeutic benefit (data not shown). Importantly, V158411 exhibited no single agent activity in either Colo205 or SW620 tumor bearing mice. The ability to administer a single IV infusion of a Chk1 inhibitor closely after administration of a cytotoxic agent could have significant patient benefits. Such a regimen would reduce the amount of time spent by the patient at the hospital and hopefully improve patient compliance. To the best of our knowledge, V158411 is the only Chk1 inhibitor under pre-clinical development with such an *in vivo* activity profile. All other inhibitors in development either require multiple doses to see efficacy or require administration 24 hours after the cytotoxic chemotherapeutic agent [27, 33, 34].

In summary, these preclinical studies demonstrate that V158411 is a potent, selective inhibitor of Chk1 that, *in vivo*, is well tolerated and enhances the anti-tumor activity of irinotecan therapy with a schedule that will be easily transferable to the clinic. These observations suggest that further clinical evaluation of V158411 is warranted.

## MATERIALS AND METHODS

### Synthesis of V158411

**Figure 7:**
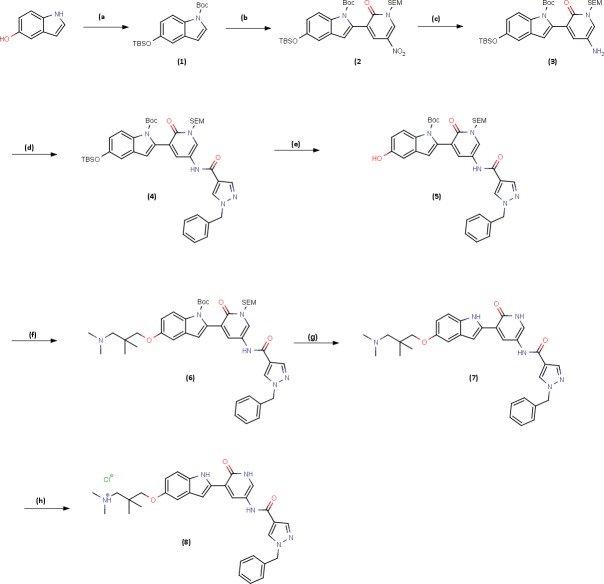
Scheme 1: Synthesis route of V158411 Reagents and conditions: **A.** TBDMSCl, DIPEA, DMAP, DCM, rt, 3 h then (BOC)_2_O, DMAP, DCM rt 2 h, 100%; **B.** LDA, (^i^PrO)_3_B, THF, 0-5°C, 30 min then Intermediate **I.**, K_2_CO_3_, Pd(dppf)Cl_2_:CH_2_Cl_2_, 60°C, 2 h, 76%; **C.** 10%Pd/C, HCO_2_NH_4_, MeOH 60°C, 1h, 100%; **D.** Intermediate (ii), Et_3_N, DCM, 0°C then rt, 18 h, 94%; **E.** TBAF, 1.0M in THF, THF, 0°C then rt, 2 h, 93%; **F.** Intermediate (iii), Cs_2_CO_3_, DMF, 100°C, 4 h, 84%, **G.** TBAF, 1.0M in THF, ethylenediamine, THF, 70°C, 18 h, 81%; **H.** HCl solution, 2.0M in Et_2_O, MeOH/CHCl_3_, 0°C then rt, 1h, 69%.

V158411 was synthesized according to Scheme 1 (Figure 7). A detailed description of the synthesis of V158411 can be found in the supplemental information.

### X-ray crystallography

X-ray crystallography was as previously described [35]. In brief, structures were obtained following soaking of apo Chk1 crystals at 18°C for 16 hours. Diffraction data was collected at the DLS synchrotron (Oxfordshire, UK) at the I02 beamline, equipped with the ADSC Q315 ccd detector. PDB ID: 5DLS (www.rcsb.org).

### Molecular modeling

Compound modelling was performed with software from CCG (http://www.chemcomp.com/) and from Schrödinger (http://www.schrodinger.com/).

### *In vitro* kinase assays

Kinase assays were performed as previously described [36].

### Cell culture and cytotoxicity assay

All cells were obtained from the American Type Culture Collection and maintained as low passage stocks. Cells were routinely cultured in DMEM or RPMI1640 containing 10% FCS and 1% penicillin / streptomycin (Invitrogen). The cytotoxicity of V158411 was determined following exposure of cells in 96 well plates using a 10-point titration for 72 hours. Cell proliferation was determined using sulphorhodamine B staining following protein precipitation with 10% TCA.

### pH2AX ELISA

1 × 10^4^ HT29 cells were seeded per well of a 96 well plate and treated with a combination of 50 nM gemcitabine plus increasing concentrations of V158411 for 24 hours. Following fixation with methanol, pH2AX was detected with a mouse monoclonal antibody (JBW301, Millipore) and a europium-labeled anti-mouse secondary antibody.

### Antibodies and western blotting

Anti-pHistone H3 (S10) was obtained from Millipore; pCdc2 (Y15), pCdc25C (S216), Chk1, pChk1 (S317), pChk2 (S516), Cyclin B1, pH2AX (S139), GAPDH and Actin from Cell Signaling Technologies and pChk1 (S296) from Abcam. Treated and untreated cells were washed once with PBS and lysed in RIPA buffer containing protease and phosphatase inhibitor cocktail (Roche). Protein concentration was determined using BCA kit (Pierce). Equal amounts of lysate were separated by SDS-PAGE and western blot analysis conducted using the antibodies indicated above.

### Potentiation assays

5 × 10^3^ cells per well were seeded in 96-well plates and incubated overnight. Cells were treated with a 10-point titration of gemcitabine, camptothecin, SN38 or cisplatin in the presence of a fixed concentration of V158411 for 72 hours. The effect on cell proliferation was determined using a CellTiter 96 AQ_ueous_ One Solution Cell Proliferation Assay (MTS, Promega).

### Clonogenicity assay

HT29 cells were plated at a density of 300 cells per well in 6-well plates and allowed to attach for 4 hours. Cells were treated with gemcitabine, camptothecin or SN38 in the absence or presence of V158411 for 24 hours followed by drug free media for a further 14 days. Resulting colonies were stained and fixed with 0.1% crystal violet in 10% formaldehyde.

### *In vivo* studies

### Animals

Animals were purchased from Charles River Laboratories or Harlan. All procedures were conducted in accordance with the Institute for Laboratory Animal Research Guide for the Care and Use of Laboratory Animals (USA) or Guidance on the Operation of the Animals (Scientific Procedures) Act 1986 (UK).

### Pharmacokinetics of V158411

Dosing of animals and collection of samples was conducted by Quotient Bioresearch (Rushden) Ltd, UK. V158411 was administered by intravenous (IV) bolus injection to female Balb/C mice, male Sprague-Dawley rats or male beagle dogs. Plasma samples were prepared by protein precipitation with acetonitrile containing internal standard (IS; dextromethorphan, 0.5 μg/mL); the analysis plate was centrifuged at 3,000 rpm for 10 minutes at 4°C. Calibration lines (1-5,000 ng/mL) were prepared in plasma for quantitation of V158411, irinotecan and SN38. Supernatant was analyzed by liquid chromatography combined with tandem mass spectrometry (LC-MS/MS) for V158411, irinotecan, SN38 and dextromethorphan. The ratios of analyte against IS peak areas were calculated, and concentrations derived from calibration lines generated by Quanlynx (Waters Ltd.).

### Calculation of pharmacokinetic parameters

Pharmacokinetic parameters were calculated using the software package WinNonLin (Pharsight Corp.). Data was analyzed using noncompartmental analysis and fitted using plasma concentration versus time curves. Irinotecan and SN38 - non-compartmental model 200, linear trapezoidal (Linear Interpolation) method; V158411 - non-compartmental model 201, linear trapezoidal (Linear Interpolation) method.

### Tolerability of V158411

Tolerability studies were undertaken by Charles River Laboratories Discovery Services, North Carolina in female HRLN *nu/nu* mice. Animals were weighed daily for the first five days then twice weekly thereafter for the duration of the study. The mice were observed frequently for overt signs of any adverse, treatment-related side effects, and clinical signs of toxicity recorded. Toxicity greater than a group mean body-weight loss of more than 20% or 1 in 10 treatment-related deaths was considered to be the maximum tolerated dose.

### Xenograft models in athymic nude mice

Xenograft studies were undertaken by Charles River Laboratories Discovery Services, North Carolina. On the day of tumor cell implant, Colo205 cells were harvested and resuspended in 50% Matrigel matrix (BD Biosciences) in PBS at a concentration of 5 × 10^6^ cells/mL. Each test mouse received 1 × 10^6^ Colo205 cells implanted subcutaneously (SC) in the flank of female athymic nude mice (HRLN or NCr *nu/nu*), and tumor growth was monitored as the average size approached 100-150 mm^3^. The SW620 tumor line was maintained by serial SC transplantation in female athymic nude mice (HRLN *nu/nu*). Tumor fragments, approximately 1 mm^3^ each, were implanted SC into the right flank of each animal and allowed to grow towards a target size of 100-150 mm^3^. On day 1 of the study, tumors were randomized into treatment groups before compound administration.

### Antitumor efficacy studies

Irinotecan (Camptostar, Pharmacia and Upjohn) was diluted in 5% dextrose in deionised water (D5W) and administered *via* the intraperitoneal (IP) route. V158411 was formulated in D5W and administered *via* IV injection into the tail vein. Cyclic treatment regimens were followed and treatment ranged from 2-3 cycles. MTD (100 mg/kg) irinotecan was administered once per week for 3 weeks in combination with V158411 2 or 24 hours after the irinotecan dose.

Tumor size was measured with electronic calipers and tumor volume calculated according to the formula ((width × width) × length) /2.

The study endpoint was defined as a tumor volume of 750 mm^3^ for Colo205 or 1500 mm^3^ for SW620, or day 60, whichever came first. Each animal was euthanized when its' tumor reached this endpoint. The time to endpoint (TTE) for each mouse was calculated from the equation TTE = (log_10(endpoint volume)_-b)/m where b is the intercept and m the slope of the line obtained by linear regression of a log-transformed tumor growth data set. Animals that did not reach the end point were assigned a TTE value equal to the last day of the study.

## SUPPLEMENTARY MATERIAL FIGURE AND TABLES


