# Supplementary material for: Identification of novel, in vivo active Chk1 inhibitors utilizing structure guided drug design
Source: Oncotarget. 2015 Sep 30;6(34):35797–812. doi: 10.18632/oncotarget.5929 (PMC4742142; doi:10.18632/oncotarget.5929)
Supplement: Supplementary file 1 [file oncotarget-06-35797-s001.pdf]

Identification of novel, *in vivo* active Chk1 inhibitors utilizing structure guided drug design

Supplementary Material

Synthesis of V158411 hydrochloride

All solvents and reagents purchased from commercial suppliers were used without further purification.

^{1}H NMR spectra were recorded on a Bruker DPX 400 MHz spectrometer in DMSO – d_6 with known chemical shift of the solvent as reference. Data for ^{1}H NMR are reported as follows: chemical shift (δ ppm), multiplicity (s = singlet, d = doublet, m = multiplet, dd = doublet of doublets and br = broad), integration and coupling constant (Hz). NMR data were analyzed using MestReNova software. High performance liquid chromatography-mass spectroscopy (HPLC-MS) spectra were recorded on an Agilent HP1100 Mass detector 1946D ESI source M/z range 150 to 1000 amu. Flash chromatography was performed with pre-packed silica-gel cartridges (Strata Si-1, 61 Å, Phenomenex, Cheshire, UK or IST Flash II, 54 Å, Argonaut, Hengoed, UK). Thin layer chromatography was conducted with 5 x 10 cm plates coated with Merck Type 60 F_{254} silica-gel. IUPAC chemical names were generated using Marvin Sketch version 15.7.20.

Step 1: Preparation of tert-butyl 5-[tert-butyldimethylsilyl)oxy]-1H-indole-1-carboxylate (1)

To a stirred solution of 5-hydroxyindole (25.0g, 188mmol) in dichloromethane (1L) at rt was added N,N-diisopropylethylamine (48.6g, 65.5mL, 376mmol) and a solution of tert-butyldimethylsilyl chloride (29.7g, 34.2mL 197mmol) in dichloromethane (100mL). 4-Dimethylaminopyridine (2.3g, 18.8mmol) was added and the reaction was stirred at rt for 3 h. The reaction mixture was concentrated *in vacuo* and the residue was taken up in ethyl acetate (500mL), washed with an aqueous 0.5N hydrochloric acid solution (400mL), brine, dried ($MgSO_4$) and concentrated *in vacuo* to give 5-[tert-butyldimethylsilyl)oxy]-1H-indole as a red oil.

This was dissolved in dichloromethane (1L) and to this was added di-tert-butyl dicarbonate (45.1g, 207mmol) as a solution in dichloromethane (100mL) drop wise at rt. 4-Dimethylaminopyridine (2.3g, 18.8mmol) was added and the reaction stirred at rt for 2 hours. The reaction mixture was concentrated *in vacuo* and the residue was taken up in ethyl acetate (500mL), washed with an aqueous 0.5N hydrochloric acid solution (400mL), brine, dried ($MgSO_4$) and concentrated *in vacuo* to afford the desired title compound as a brown oil, 65.3g, 100%.

Step 2: Preparation of tert-butyl 5-[(tert-butyldimethylsilyl)oxy]-2-(5-nitro-2-oxo-1-{[2-(trimethylsilyl)ethoxy]methyl}-1,2-dihydropyridin-3-yl)-1H-indole-1-carboxylate (2)

To a solution of diisopropylamine (11.3g, 15.6mL, 112mmol) in anhydrous tetrahydrofuran (100mL) was added butyllithium solution 2.5M in hexanes (41.6mL, 104mmol) drop wise at -78°C. After addition the reaction mixture was allowed to attain 0°C, where it was stirred for 30 minutes to form the lithiumdiisopropyl amide solution.

Triisopropyl borate (22.8g, 28.0mL, 121mmol) was added to a solution of tert-butyl 5-[tert-butyldimethylsilyl)oxy]-1H-indole-1-carboxylate (30.5g, 88mmol) in tetrahydrofuran (1L). The reaction mixture was cooled to -5°C and the previously described lithiumdiisopropyl amide solution was added drop wise keeping the temperature between -5°C and 0°C. After addition the reaction was stirred at 0°C for 30mins and then the reaction mixture was concentrated *in vacuo* to afford crude {1-[(tert-butoxy)carbonyl]-5-[(tert-butyldimethylsilyl)oxy]-1H-indol-2-yl}boronic acid, which was dissolved in tetrahydrofuran (1L). To this was added water (160mL), potassium carbonate (33.1g, 239mmol), Intermediate i, 3-iodo-5-nitro-1-(2-trimethylsilanyl-ethoxymethyl)-1H-pyridin-2-one, (31.6g, 80mmol) and [1,1'-bis(diphenylphosphino)ferrocene)]dichloropalladium(II), complex with dichloromethane (3.3g, 5mol%). The red suspension was degassed for 10 minutes and then heated at 60°C for 2 hours. The reaction mixture was cooled and was partitioned between ethyl acetate (500mL) and aqueous saturated sodium bicarbonate solution (400mL). The organic layer was separated and the aqueous was extracted with a further portion of ethyl acetate. The combined ethyl acetate layers were washed with brine, dried ($MgSO_4$), filtered through a plug of celite and concentrated *in vacuo*. The resultant crude product was purified by flash chromatography on SiO_2 eluting with hexane – 30% ethyl acetate / hexane (gradient). The fractions containing product were combined, concentrated *in vacuo* and the residue was triturated using iso-hexane to afford the title compound as a pale yellow solid, 37.2g, 76%.

Step 3: Preparation of tert-butyl 2-(5-amino-2-oxo-1-{[2-(trimethylsilyl)ethoxy]methyl}-1,2-dihydropyridin-3-yl)-5-[(tert-butyldimethylsilyl)oxy]-1H-indole-1-carboxylate (3)

To a suspension of tert-butyl 5-[(tert-butyldimethylsilyl)oxy]-2-(5-nitro-2-oxo-1-{[2-(trimethylsilyl)ethoxy]methyl}-1,2-dihydropyridin-3-yl)-1H-indole-1-carboxylate (30g, 48.7mmol) and ammonium formate (46.0g, 729mmol) in MeOH (500mL) was added palladium, 10% on carbon, (0.6g). The suspension was degassed and then heated at 60°C for 1 h and then allowed to cool to RT. The mixture was filtered through celite, concentrated *in vacuo* and the residue taken up in ethyl acetate (600mL). The organics were washed with water (4x400mL), brine (2x400mL), dried ($MgSO_4$) and concentrated *in vacuo* to give the title compound as a dark green foam, 28.5g, 100%.

Step 4: Preparation of tert-butyl 2-[5-(1-benzyl-1H-pyrazole-4-amido)-2-oxo-1-{[2-(trimethylsilyl)ethoxy]methyl}-1,2-dihydropyridin-3-yl]-5-[(tert-butyldimethylsilyl)oxy]-1H-indole-1-carboxylate (4)

To a solution of tert-butyl 2-(5-amino-2-oxo-1-{[2-(trimethylsilyl)ethoxy]methyl}-1,2-dihydropyridin-3-yl)-5-[(tert-butyldimethylsilyl)oxy]-1H-indole-1-carboxylate (28.5g, 48.7mmol) in DCM (400mL) was added trimethylamine (19.7g, 27.1mL, 195mmol) and the mixture cooled to 0°C. A solution of Intermediate ii, 1-benzyl-1H-pyrazole-4-carbonyl chloride (12.9g, 58.4mmol) in DCM (100mL) was added drop wise over 20 min. After addition the reaction mixture was allowed to attain rt over 30 min and then stirred at rt for 18 h. The reaction mixture was washed with saturated aqueous sodium hydrogen carbonate (500mL), brine (400mL), dried ($MgSO_4$) and concentrated *in vacuo*. The residue was triturated with iso-hexane to give a pink solid that was further purified by flash chromatography on SiO_2 eluting with hexane – 60% ethyl acetate / iso-hexane (gradient). The fractions containing pure product were combined, concentrated *in vacuo* and triturated with diethyl ether to afford the title compound as 26.016 g, 69% as a white solid, 26.0g, 69%. The mixed fractions containing product were combined, concentrated *in vacuo* and triturated with diethyl ether to give a second batch of the title compound as a white solid, 4.9g, 13%. The filtrates from all the diethyl ether triturations were combined and concentrated *in vacuo*. The residue was purified by flash chromatography on SiO_2 eluting with hexane – 60% ethyl acetate / iso-hexane (gradient). The fractions containing product were combined, concentrated *in vacuo* and triturated with iso-hexane to afford a third batch of the title compound as pink solid, 4.4g, 12%. The total yield for this step was 35.3g, 94%.

Step 5: Preparation of tert-butyl 2-[5-(1-benzyl-1H-pyrazole-4-amido)-2-oxo-1-{[2-(trimethylsilyl)ethoxy]methyl}-1,2-dihydropyridin-3-yl]-5-hydroxy-1H-indole-1-carboxylate (5)

To a stirred solution of tert-butyl 2-[5-(1-benzyl-1H-pyrazole-4-amido)-2-oxo-1-{[2-(trimethylsilyl)ethoxy]methyl}-1,2-dihydropyridin-3-yl]-5-[(tert-butyldimethylsilyl)oxy]-1H-indole-1-carboxylate (30.9g, 40.2mmol in tetrahydrofuran (300mL) at 0°C was added tetrabutylammonium fluoride solution, 1.0M in tetrahydrofuran (42.2mL, 42.2mmol) drop wise. After addition the reaction was stirred at 0°C for a further 10 min and then allowed to attain rt, where it was stirred for a further 2 h. The reaction mixture was diluted with ethyl acetate (300mL), washed with water (300mL), aqueous 0.5N hydrochloric acid solution (2x100mL), brine, dried ($MgSO_4$) and concentrated *in vacuo*. The residue was triturated several times using diethyl ether to give the title compound as a white solid, 24.5g, 93%.

Step 6: Preparation of tert-butyl 2-[5-(1-benzyl-1H-pyrazole-4-amido)-2-oxo-1-{[2-(trimethylsilyl)ethoxy]methyl}-1,2-dihydropyridin-3-yl]-5-[3-(dimethylamino)-2,2-dimethylpropoxy]-1H-indole-1-carboxylate (6)

To a solution of tert-butyl 2-[5-(1-benzyl-1H-pyrazole-4-amido)-2-oxo-1-{[2-(trimethylsilyl)ethoxy]methyl}-1,2-dihydropyridin-3-yl]-5-hydroxy-1H-indole-1-carboxylate (2.43g, 3.7mmol) in N,N-dimethylformamide (100mL) was added Intermediate iii, (3-chloro-2,2-dimethylpropyl) dimethylamine hydrochloride (1.38g, 7.4mmol) and cesium carbonate (3.62g, 11.1mmol). The reaction mixture was heated with stirring at 100°C for 4 h and cooled to RT. The mixture was filtered and the filtrate diluted with H_20 (100mL) and extracted with ethyl acetate (2x100mL). The combined extracts were washed with brine (4x200mL), dried ($MgSO_4$) and concentrated *in vacuo.* The residue was purified by flash chromatography on SiO_2 eluting with dichloromethane – 10% methanol / dichloromethane (gradient). The fractions containing product were combined and concentrated *in vacuo*. The residue was triturated with iso-hexane to afford the desired title compound as a pale yellow solid, 2.4g, 84%.

Step 7: Preparation of V158411, 1-benzyl-N-(5-{5-[3-(dimethylamino)-2,2-dimethylpropoxy]-1H-indol-2-yl}-6-oxo-1,6-dihydropyridin-3-yl)-1H-pyrazole-4-carboxamide (7)

To a solution of tert-butyl 2-[5-(1-benzyl-1H-pyrazole-4-amido)-2-oxo-1-{[2-(trimethylsilyl)ethoxy]methyl}-1,2-dihydropyridin-3-yl]-5-[3-(dimethylamino)-2,2-dimethylpropoxy]-1H-indole-1-carboxylate (12.7g, 16.5mmol) in tetrahydrofuran (250mL) was added 1,2-diaminoethane (4.96g, 5.5ml, 82.5mmol) and tetrabutylammonium fluoride solution, 1.0M in tetrahydrofuran (82.5mL, 82.5mmol) and the mixture heated at 70°C for 18 h. After cooling, the reaction mixture was poured into a mixture of H_20 (750mL) and MeCN (375mL) that had been cooled to 0°C. The mixture was stirred for 1 h at this temperature and then the solids were collected via filtration. The yellow solid collected was washed with water (4 x 100 ml), MeCN (4 x 100 ml) ether (2 x 150 ml) and more MeCN (100 mL) to afford the title compound as a yellow solid, 7.2g, 81%.

LC/MS: RT = 1.73 Min (270nm), *m/z* = 539 [M+H]. Total run time 3.75 min (short pos).

^{1}H NMR (400 MHz, DMSO-d_6) δ 12.01 (br s, 1H), 11.46 (br s, 1H), 9.81 (s, 1H), 8.44 (s, 1H), 8.17 (d, *J* = 2.8 Hz, 1H), 8.06 (s, 1H), 7.87 (d, *J* = 2.7 Hz, 1H), 7.46 – 7.27 (m, 6H), 7.04 (d, *J* = 2.4 Hz, 1H), 6.94 (d, *J* = 2.1 Hz, 1H), 6.75 (dd, *J* = 8.8, 2.4 Hz, 1H), 5.43 (s, 2H), 3.70 (s, 2H), 2.27 (s, 2H), 2.23 (s, 6H), 0.98 (s, 6H).

Step 8: Preparation of V158411 HCl salt, 1-benzyl-N-(5-{5-[3-(dimethylamino)-2,2-dimethylpropoxy]-1H-indol-2-yl}-6-oxo-1,6-dihydropyridin-3-yl)-1H-pyrazole-4-carboxamide hydrochloride salt (8)

To a solution of 1-benzyl-N-(5-{5-[3-(dimethylamino)-2,2-dimethylpropoxy]-1H-indol-2-yl}-6-oxo-1,6-dihydropyridin-3-yl)-1H-pyrazole-4-carboxamide (7.2g, 13.3mmol)) in a mixture of $CHCl_3$ (200mL) and MeOH (15mL) was added a hydrogen chloride solution, 2.0 M in diethyl ether (20mL, 20mmol) drop wise 0°C. After addition, stirring was continued for one hour as the reaction was allowed to attain rt. The suspension was poured onto a mixture of stirring MeOH (25mL) and MeCN (225mL) and stirred for 1 hour before collecting the yellow solid by filtration. The solids were washed several times with 10% MeOH in MeCN, and dried *in vacuo* to give the title compound, V158411 hydrochloride salt as a yellow solid, 5.3g, 69%.

LC/MS: RT = 1.71 Min (270nm), *m/z* = 539 [M-HCl]. Total run time 3.75 min (short pos).

^{1}H NMR (400 MHz, DMSO-d_6) δ 12.03 (br s, 1H), 11.50 (d, *J* = 2.3 Hz, 1H), 9.90 (s, 1H), 9.33 (br s, 1H), 8.46 (s, 1H), 8.24 (d, *J* = 2.8 Hz, 1H), 8.08 (s, 1H), 7.88 – 7.82 (m, 1H), 7.49 – 7.26 (m, 6H), 7.09 (d, *J* = 2.4 Hz, 1H), 6.95 (d, *J* = 1.9 Hz, 1H), 6.81 (dd, *J* = 8.8, 2.3 Hz, 1H), 5.42 (s, 2H), 3.87 (s, 2H), 3.25 (d, *J* = 4.2 Hz, 2H), 2.85 (d, *J* = 4.8 Hz, 6H), 1.18 (s, 6H).

Preparation of Intermediate: (i) 3-Iodo-5-nitro-1-(2-trimethylsilanyl-ethoxymethyl)-1H-pyridin-2-one

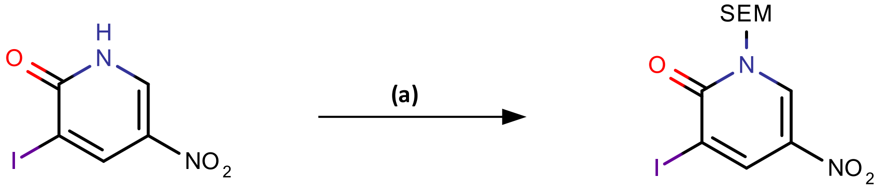

INTERMEDIATE (i)

Reagents and conditions: (a) SEMCl, LiOH.H2O, 1,2-DME, 5°C then rt, 2 h, 68%

2-Hydroxy-3-iodo-5-nitro pyridine (26.6g, 100mmol) was stirred in anhydrous 1,2-dimethoxyethane (500ml) at ambient temperature and lithium hydroxide monohydrate (8.5g, 203mmol) was added. The reaction mixture was cooled to 5°C using ice/water and then 2-(Trimethylsilyl)ethoxymethyl chloride (18.84g, 20ml, 113mmol) was added drop wise and the reaction stirred at ambient temperature for 2 hours. The reaction mixture was diluted with diethyl ether (1000ml) and the mixture washed with 2M aqueous sodium hydroxide solution (2x200ml) and then 20% aqueous sodium thiosulphate solution (200ml) and finally water (3x200ml). The solution was dried over anhydrous sodium sulphate and concentrated to an orange oil. The resultant crude product was purified by flash chromatography on SiO_2 eluting with hexane - 20% ethyl acetate / hexane

(gradient). The fractions containing pure product were combined and concentrated *in vacuo* to afford the title compound as a yellow oil, 26.9g, 68%.

Preparation of Intermediate (ii): 1-Benzyl-1H-pyrazole-4-carbonyl chloride

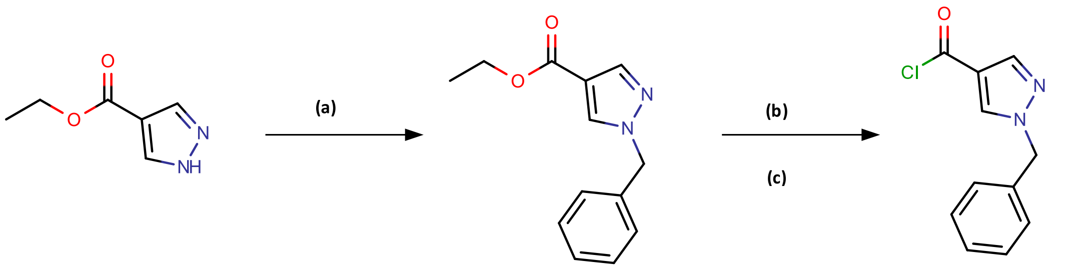

INTERMEDIATE (ii)

Reagents and conditions: (a) BnBr, K_2CO_3, acetone, 50°C, 18 h, 100%; (b) KOH, MeOH/H_2O, 100°C, 3h, 93%; (c) $SOCl_2$, $PhCH_3$, 110°C, 3 h, 100%.

1H-Pyrazole-4-carboxylic acid ethyl ester (10.0g, 71.4mmol) was stirred in acetone (300mL) with potassium carbonate (29.6g, 214.2mmol) and benzyl bromide (14.4g, 9.33mL, 78.5mmol) was added. The reaction was heated at 50°C for 18 hours. The reaction was cooled and the inorganics were separated via filtration. The filter cake was washed through with ethyl acetate (2x100mL) and the combined washings and filtrate were concentrated *in vacuo* to give ethyl 1-benzyl-1H-pyrazole-4-carboxylate as a red oil, 16.4g, 100%.

Ethyl 1-benzyl-1H-pyrazole-4-carboxylate (16.4g, 71.4mmol) was taken up in a mixture of methanol (250mL) and H_2O (50mL). Potassium hydroxide (8g, 145mmol) was added and the mixture heated at reflux for 3 h. The heating was removed and once cooled the reaction mixture was concentrated *in vacuo*. The residue was taken up in H_2O (300mL) and washed with ethyl acetate (2x200mL) and the combined organic layers discarded. The basic aqueous layer was acidified to pH4 using an aqueous 6N hydrochloric acid solution. The resulting precipitate was filtered, washed with copious amounts of water and dried *in vacuo* to afford 1-benzyl-1H-pyrazole-4-carboxylic acid as a white solid, 13.4g, 93%.

1-Benzyl-1H-pyrazole-4-carboxylic acid (11.8g, 58.4mmol) was stirred in toluene (200mL) at RT and thionyl chloride (13.9g, 8.5mL, 116.8mmol) was added drop wise. The reaction mixture was slowly heated to reflux and held there for 3 h. After cooling the reaction was concentrated *in vacuo*. Toluene (50mL) was added to the residue and concentrated *in vacuo*. This was repeated a further three times with toluene (50mL) and then with iso-hexane (50mL) to give the title compound as a yellow oil, 12.9g, 100%.

Preparation of Intermediate (iii): (3-chloro-2,2-dimethylpropyl) dimethylamine hydrochloride

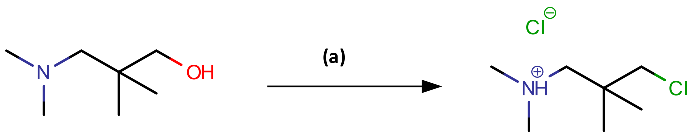

INTERMEDIATE (iii)

Reagents and conditions: (a) $SOCl_2$, $PhCH_3$, DMF, 110^oC, 3 h, 94%.

To a solution of 3-(dimethylamino)-2,2-dimethylpropan-1-ol (5.0g, 38.1mmol) in toluene (100ml) containing DMF (0.1mL) was added thionyl chloride (5.0g, 3.1mL, 41.9mmol) drop wise. After addition the reaction mixture was slowly heated to reflux and held there for 3 h. After cooling the reaction was concentrated *in vacuo*. Toluene (50mL) was added to the residue and the mixture was sonicated for 10 min. The solids were separated via filtration and washed with toluene and iso-hexane to afford the title compound as a light brown solid, 6.7g, 94%.

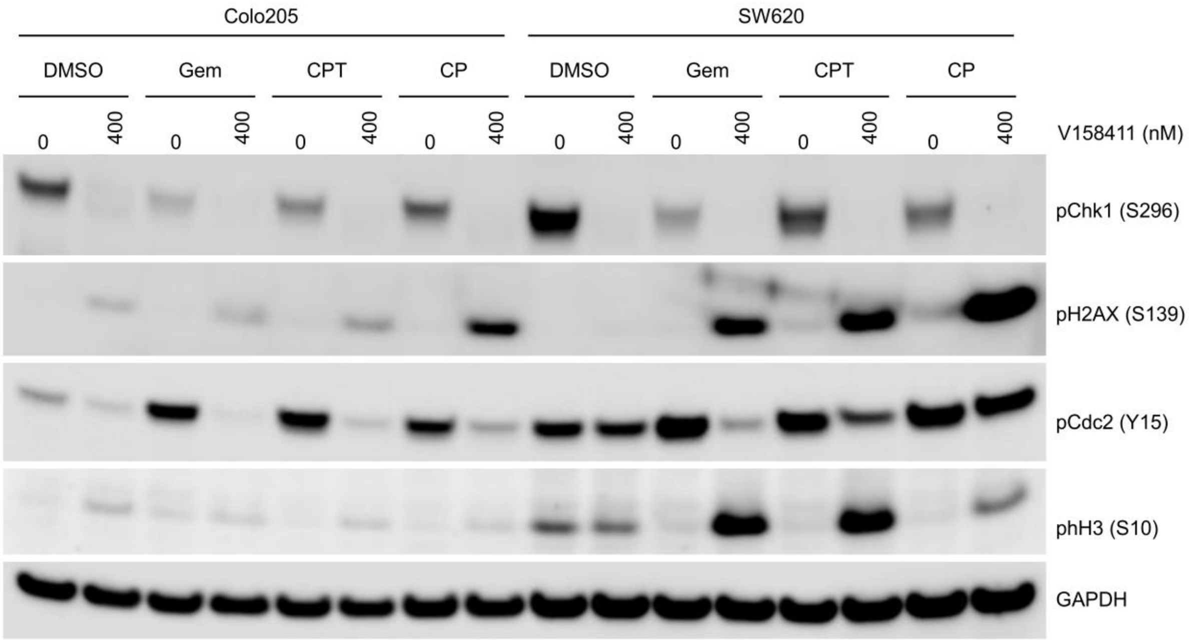

Supplementary Figure 1. Effect of V158411 on gemcitabine and camptothecin induced DNA damage checkpoint and cell cycle proteins in Colo205 and SW620 cells

Colo205 or SW620 cells were treated with gemcitabine (125 nM), camptothecin (250 nM) or cisplatin (30 µM) for 2 hours followed by V158411 (400 nM) for a further 24 hours. Protein expression was characterised by immunoblotting as described in materials and methods.

Supplementary Table 1. *In vitro* kinase selectivity of V158411

Percent inhibition at 50 nM	Kinase
99 – 100	Chk1 (100% inhibition at this concentration)
90 – <99	AAK1, RIOK1, RIOK3
65 – <90	BIKE, FLT3, GRK4, LKB1, LOK, MEK5, MELK, PDGFRB, PIP5K1A, PIP5K2B, PKN1, PRKG2, PRKX, SLK, SNARK, SRPK1, SRPK3, STK16, YSK4

Supplementary Table 2. Cellular activity of V158411 in human cancer cell lines

Cell Line	Tissue	GI_{50} (μM)
Colo205	Colon	1.6 ± 0.42
HCT116	Colon	4.0 ± 0.78
HT29	Colon	0.65 ± 0.25
SW620	Colon	0.50 ± 0.033
A549	Lung	1.4 ± 0.35
NCI-H460	Lung	2.7 ± 0.93
NCI-H520	Lung	0.55 ± 0.14
PC3	Prostate	9.5 ± 1.0

Values are the average of at least 4 independent determinations ± SD
